# Diabetic Retinopathy: From Animal Models to Cellular Signaling

**DOI:** 10.3390/ijms23031487

**Published:** 2022-01-27

**Authors:** Priyamvada M. Pitale, Marina S. Gorbatyuk

**Affiliations:** 1 Department of Ophthalmology, Baylor College of Medicine, Houston, TX 77030, USA; priyamvada.pitale@bcm.edu; 2 Department of Optometry and Vision Science, School of Optometry, University of Alabama at Birmingham, Birmingham, AL 35294, USA

**Keywords:** animal models of diabetes, diabetic retina, electrophysiology of diabetic retina, cellular signaling of diabetic retina, tribbles homolog 3 protein

## Abstract

Diabetic retinopathy (DR) is an ocular complication of diabetes mellitus (DM), a metabolic disorder characterized by elevation in blood glucose level. The pathogenesis of DR includes vascular, neuronal, and inflammatory components leading to activation of complex cellular molecular signaling. If untreated, the disease can culminate in vision loss that eventually leads to blindness. Animal models mimicking different aspects of DM complications have been developed to study the development and progression of DR. Despite the significant contribution of the developed DR models to discovering the mechanisms of DR and the recent achievements in the research field, the sequence of cellular events in diabetic retinas is still under investigation. Partially, this is due to the complexity of molecular mechanisms, although the lack of availability of models that adequately mimic all the neurovascular pathobiological features observed in patients has also contributed to the delay in determining a precise molecular trigger. In this review, we provide an update on the status of animal models of DR to help investigators choose an appropriate system to validate their hypothesis. We also discuss the key cellular and physiological events of DR in these models.

## 1. Introduction

Diabetic retinopathy (DR) is known to be an eye complication of diabetes mellitus (DM). If untreated, it can threaten the vision of affected individuals. Current clinical trials using in vivo imaging techniques have reported dramatic retinal morphological changes associated with diabetes. A study with 124 human subjects in the early stage of DR reported a decrease in the thickness of the nerve fiber layer (NFL) with no effects to the outer neural layer (ONL) of the retina, measured by spectral domain optical coherence tomography (SD-OCT) [1]. Electroretinographic changes have also confirmed retinal dysfunction in patients with severe ocular diabetic complications [2,3]. Furthermore, changes in retinal hemodynamics have also been reported in patients with early DM [4]. Currently, DR is recognized as a progressive neuro-vascular complication with neuronal dysfunction proceeding to microvascular damage [5]. The early stage of the disease is known as non-proliferative diabetic retinopathy (NPDR); it ranges from mild (microaneurysms) to severe (decrease in the blood flow due to blockade in a larger section of retinal blood vessels). Proliferative diabetic retinopathy (PDR), an advanced stage in which blood vessels grow in the retina, often leads to a reduced field of vision and blindness. While clinical trials concentrate on risk factors, early detection, and evaluations of the progression of DR in vivo, access to human donor eye tissue provides a great opportunity to study early molecular changes in the diabetic retina to further understand pathological markers. Multiple studies with postmortem donor eyes have reported glial cell dysfunction as a primary change in the diabetic retina. Thus, a recent study with postmortem diabetic ocular tissue that employed an immunolabelling technique to detect carbonic anhydrase (II) and glial fibrillary acidic protein (GFAP) identified the occurrence of Müller cell reactivation in the human diabetic retina [6,7]. The authors discovered that the Müller cells migrated in the pre-retinal membranes and overexpressed GFAP in the diabetic donor eyes.

In addition to structural and morphological alterations, molecular changes occurring in diabetic retinas have also been reported. Thus, studies on post-mortem diabetic eyes have shown the elevation of inflammatory markers; an increase in pro-death caspase-3, Fas, and Bax in the retinal ganglion cells (RGC); and GFAP in the retina [8,9,10]. Several studies on vitreous extracted from patients demonstrated that levels of interleukin-8, monocyte chemotactic protein-1, macrophage-colony stimulating factor, platelet-derived growth factor (PDGF), and vascular endothelial growth factor (VEGF) are elevated compared with non-diabetic individuals [11,12,13,14]. In addition, extracellular matrix proteins and an elevated expression of genes associated with angiogenesis and apoptosis were identified in fibrovascular membranes extracted from PDR patients during vitrectomy [15]. These analyses have also helped to identify potential therapeutic targets. Thus, it has been confirmed that VEGF plays an important role in the development of aberrant neovascularization in diabetic retinas and that VEGF is a biomarker of microangiopathy in PDR [16]. In addition to VEGF, increases in the number of apoptotic cells measured by a terminal deoxynucleotidyl transferase-mediated dUTP nick end labeling (TUNEL) assay, as well as pericyte and endothelial cell loss, were reported in the retinas of patients with diabetic microvascular complications [17]. Although studies with human donor tissues are an excellent asset for improving our understanding of the molecular signaling contributing to DR pathobiology, they cannot provide a complete picture of the mechanism of the development of DR. Moreover, human donor tissues may not be readily available. The use of genetic animal models addresses these limitations, and they provide an excellent approach to developing a comprehensive understanding of the cellular pathways associated with DR. While the choice of the appropriate animal model that mimics all aspects of human DR pathology is challenging, several models can capture key cellular and physiological events of diabetic retinopathy in humans. Therefore, in this literature review, we summarize the current status of the development of animal models used in research focusing on diabetic retinopathy.

## 2. Experimental Diabetic Retinopathy

One of the key regulators of homeostatic balance within the glucose metabolism in the body is insulin, which is produced by the beta cells of the pancreas. The insulin receptor–signaling pathway facilitates the entry of glucose into the cells through the activation of the protein kinase B (AKT)-mediated glucose transporter (GLUT1). In humans, fasting blood glucose level (BGL) is maintained in the range of 92–126 mg/dL, while the postprandial blood glucose level is in the range of 97–140 mg/dL. It is well accepted that under fasting and postprandial conditions, BGL levels above 126 mg/dL and 180 mg/dL, respectively, are considered sustained hyperglycemia [18]. It is now accepted that hyperglycemia primarily drives NPDR, while sustained hypoxia results in the progression of PDR. Thus, based on these facts, animal models that mimic the pathophysiological events of DR were developed. These models differ by the approaches used to induce hyperglycemia (pharmaceutical agents, pancreatectomy, or genetically modified animals [19,20,21,22,23]), their classification in the phylogenic tree (rat, mouse, rabbit, monkey, zebrafish, dog, pig, cat, and tree shrew), the observed neuronal and vascular changes, and the activation of cellular signaling (Table 1).

### Induction of Hyperglycemia

The pharmacological induction of hyperglycemia with streptozotocin (STZ) is the method used most frequently to develop a type 1 diabetes (T1D) model. Antibiotic STZ is produced by the bacterium *Streptomyces achromogens* and possesses a broad spectrum of antibacterial properties. Highly reactive methyl nitrosourea moiety is responsible for its cytotoxic effect, resulting in pancreatic β cell necrosis, whereas glucose moiety facilitates its transports to the pancreatic β cells. STZ acts via the GLUT2 receptors abundantly present on β cell plasma membranes, which make pancreatic β cells a specific target of STZ [19]. When administered either on five consecutive days or as a single dose, STZ leads to hyperglycemia [24]. For example, it has been reported that the STZ dosage for multiple intraperitoneal (IP) injections ranges from 40 to 80 mg/kg body weight (bw). A single dose administered within the range of 150–200 mg/kg bw in mice or 30–100 mg/kg bw in rats by IP injection also induces hyperglycemia [25,26,27,28]. In rabbits, intravenous injection (IV) with a dose of 110 mg/kg bw has been reported to trigger hyperglycemia [29]. In contrast, a single dose of 175 mg/kg is required to induce hyperglycemia in tree shrews [31]. Interestingly, the maintenance of fasting or non-fasting conditions before STZ injection does not change the postinduction hyperglycemic effect of STZ [62]. The hyperglycemia after STZ injection is usually seen within one to four weeks in most species. In some cases, insulin injections are necessary for hyperglycemic mice and rats to control the extreme fluctuations in the BGL, although they are not always necessary for STZ models [24]. An alternative to STZ, Alloxan, can also be used to induce hyperglycemia. This drug is commonly used in mice, rats, rabbits, and pigs. A pyrimidine derivative, alloxan, directly targets the beta cells of the pancreas, causing apoptosis by inhibiting the enzyme glucokinase and subsequently increasing the BGL due to lack of insulin production [21,35]. In rodent models, alloxan-induced hyperglycemia can be developed within one week of administration, while it takes less than a day in dogs [32].

Surgical and diet-induced hyperglycemia are alternative methods to induce experimental hyperglycemia. For example, in canine models, hyperglycemia develops three to four weeks after the surgery [35,36]. To accelerate the induction of hyperglycemia, the pharmacological approach can be combined with pancreatectomy. In addition to the above methods, dietary modifications can cause changes in the BGL. A high glucose/galactose diet is one such approach. Thus, Engerman and Kern used a high galactose diet to induce DR in dogs [22]. Other researchers have documented that diet-induced hyperglycemia leads to the development of DR in mice and rats [35]. However, it is worth mentioning that this approach can take years to develop DR in dogs and monkeys, while in rodents, the development of DR occurs much faster [22,38]. Genetic models of hyperglycemia were generated in rodents and zebrafish carrying gene mutations that lead to spontaneous hyperglycemia. These models are relatively easy to work with, and economical to develop and inbreed in controlled environments.

## 3. Rodent Models of Diabetic Retinopathy

Rat models. There are several spontaneous hyperglycemic rat models, including bio-breeding (BB) rats developing T1D, Wistar Bonn/Kobori (WBN/Kob), Zucker diabetic fatty (ZDF), Otsuka Long-Evans Tokushima fatty (OLETF), and spontaneous diabetic Torii (SDT) rats developing T2D. The BB rats manifest autoimmune DM and DR based on hyperglycemia registered at three months of age and retinal vascular changes at 8–11 months [48,49]. In WBN/Kob male rats, the onset of hyperglycemia occurs at nine months of age [50]. In contrast, ZDF rats develop hyperglycemia earlier, between 5 and 10 weeks of age. These animals are considered a non-insulin-dependent DM model. They are obese and carry a missense mutation known as *(fa/fa)* mutation in the leptin receptor gene (Lepr). Originally, these rats were derived from the Zucker rats, which are an obesity disease model [51,52]. Male OLETF rats develop high BGL starting at five months [53]. In the SDT rat model, detection of glucose in the urine, which is a common sign of glucosuria and kidney damage in patients, was reported at 20 weeks of age in males and at 45 weeks of age in females [54].

Mouse models. Ins2^Akita^, non-obese NOD, Kimba and Akimba mice developing T1D, and db/db mice developing T2D are the most popular genetic models of DM. Ins2^Akita^ mice have a point mutation in *insulin2* (earlier reported locus *Mody4*), which causes abnormal insulin production by the pancreatic cells, leading to pancreatic cell death. The heterozygous Ins2^Akita^ males are progressively hyperglycemic starting at four weeks of age, while females exhibit mild symptoms of DM. They have an average life span of 305 days and are primarily a model of early retinal complications caused by diabetes in humans [41,42]. Another model of T1D is the NOD mouse. These mice mimic human autoimmune insulin-dependent DM and exhibit CD4 and CD8 T cell-mediated autoimmune destruction of the pancreas [43,44]. Interestingly, there is a gender-based variability in the timeline for the development of hyperglycemia in these mice. Thus, 80% of the NOD females develop hyperglycemia at the age of 12 weeks, while males develop hyperglycemia later, at around 20 weeks of age [44]. The recently developed Kimba mice are a transgenic line (tr029VEGF) that mimics both NPDR and mild PDR [46]. This model is used for breeding with the Ins2^Akita^ mice to generate an Ins2^Akita^/VEGF^+/−^genotype and is known to be a new model for the comprehensive study of the mechanism of DR as a complication of T1D [47]. Another model, homozygous for the mutation (Lepr^db^) db/db mice, manifests signs of T2D and develops hyperglycemia at the age of 8–10 weeks (300 mg/dL B6.BKS(D)-*Lepr^db^*/J, stock#000697) and at the age of 6 weeks (300 mg/dL, BKS.Cg-Dock7^m^ +/+ *Lepr^db^*/J, stock# 000642). These mice are widely used because, in addition to hyperglycemia, they model obesity and metabolic disorders [45].

### 3.1. Pathological Signs in Rodent Models of Diabetic Retinopathy

#### 3.1.1. Neovascularization and Microvascular Changes in Diabetic Rodents

The most critical pathologic findings of PDR are neovascularization, hemorrhage, and fibro-vascular proliferation, leading to retinal traction and detachment and vitreous hemorrhage [63]. Oxygen-induced retinopathy (OIR) in rodents is an accurate and reproducible model of vascular proliferative changes in the retina [55]. Hypoxia-driven vascular proliferative changes seem to be similar to those seen in the retinopathy of prematurity, age-related macular degeneration, and diabetic retinopathy. OIR was developed in canine models for the first time in the early 1950s. In this model, Arnall Patz and colleagues investigated the effects of hyperoxia on retinal vessel development to study proliferative retinopathy [56,57]. To develop this model, one-day-old pups were exposed to hyperoxia for four consecutive days. In the early 1990s, this approach was introduced in rodents by Dr. Smith and her colleagues and has gained increasing popularity. In addition to OIR canines and rodents, aberrant angiogenesis has also been reported in zebrafish, rabbit, and monkey models.

The rodent OIR model is the most common approach to investigating the effect of hypoxia on the retina since it mimics the characteristics of human retinal proliferative changes [55,58,64]. Because retinal vasculature develops in the first two weeks of birth in rodents, researchers can leverage this opportunity to analyze the aberrant vascular development triggered by hypoxia. In this model, hypoxia is induced at postnatal day (P) 7 after the regression of hyaloid vessels to avoid the development of mixed hyaloidopathy. The rodent pups were then exposed to hyperoxia (75% oxygen) for five consecutive days from P7 to P12 and then observed at room air from P13 to P17 [55]. The peak changes of neovascularization are usually observed at P17, and these are resolved by P25. The C57BL/6 mice or the Sprague Dawley (SD) rats are the common strains employed in this model due to their neovascular susceptibility to hypoxia [58,64,65]. The OIR mice developed irregular blood vessels and a reduction in the retinal inner and deep plexuses at P18, mimicking retinal proliferative events triggered by hypoxia in patients with diabetic complications [66]. Downie and colleagues reported an increase in extraretinal neovascularization and impaired pericyte distribution in the OIR SD rat retinas as early as P18 [67].

Genetically modified Akimba, Akita, and Kimba mice manifest vascular dysfunction. Akimba mice were specifically developed to study the microvascular changes of DR and showed these changes at the early age of eight weeks old [47]. Thus, at eight months of age, these mice developed neovascularization, retinal edema, and detachment that progressed further through 25 weeks of age [47]. In the Kimba mice, abnormal blood vessel development was seen as early as P28, while an increase in vascular permeability and adherent leukocytes was observed at six weeks of age. Additionally, loss of retinal capillaries, neovascularization, an increased avascular area, alteration in the vessel length, and pericyte loss were reported from nine weeks to the advanced age of 24 weeks [46,68]. Vascular dysfunction in Ins2^Akita^ mice presents as an increase in leukocytosis at eight weeks, compromised vascular permeability at 12 weeks, microaneurysms at six months, and neovascularization at nine months of hyperglycemia [42,69].

STZ mice also show microvascular changes earlier in the course of diabetes compared to STZ-induced hyperglycemic rats. For example, vascular permeability detected by imaging the distribution of fluorescein-conjugated dextran is compromised in these animals as early as eight days post-STZ injection [70]. However, a decrease in arteriolar diameter and velocity were reported at four weeks and eight weeks post-STZ injection, respectively [26]. Later in the course of diabetes (six to nine months), the STZ-induced hyperglycemic mice manifested pericyte loss and developed acellular capillaries [71].

In albino Wistar–Kyoto rats, the blood retinal barrier (BRB) disruption occurs as early as two weeks post-STZ injection. Several studies reported early neovascular changes such as adherent leukocytosis and thickening of the basement membrane occurring at 8 and 12 weeks, respectively [8,72,73]. Gong et al. noted that neovascularization in STZ-injected SD rats can be observed at three to four months after induction of hyperglycemia. An increase in VEGFR1 and VEGFR2 expression levels was associated with neovascularization in STZ-induced rats [74]. Similar findings were observed in the Alloxan-induced diabetic rats; leukocytosis and neovascularization were reported at two and nine months after induction of hyperglycemia, respectively. At two months of sustained hyperglycemia, the authors observed pericyte loss, the formation of acellular capillaries, and basement membrane thickening [75,76]. In contrast, several other studies reported that BB rats with autoimmune T1D manifested these retinal changes as early as four months, while this model as well as genetic ZDF and obese OLETF rat models demonstrated BRB breakdown and pericyte loss at six to eight months [48,49,51,53,77,78]. Overall, these studies imply that the observed vascular dysfunction could vary in rat models of DR triggered by different insults. In addition to rats, hyperglycemia induced by a high-fat diet in db/db mice with T2D also leads to an increase in vascular permeability and basement thickness at 13–14 weeks of hyperglycemia [79,80]. Moreover, these mice also demonstrate pericyte loss, blood retinal barrier disruption, and vascular leakage at 12 months of age [39].

Neuronal cell death and gliosis are observed in the diabetic retina of animals with diabetes. Thus, in hyperglycemic rats, GFAP activation has been reported. STZ injection results in an increase in GFAP immunoreactivity in the retina as early as six to seven weeks [81] and as late as 8–16 weeks post-injection [81,82]. Retinal cell loss and functional changes have also been reported as early as two weeks and as late as 24 weeks post-STZ injection. Moreover, an increase in apoptotic cell death in the ONL, INL, and RGC layers resulting in a decrease in the total retinal thickness has been detected between 12 and 16 weeks post-STZ injections in rats [82,83]. In contrast, necrotic RGC death was reported at four weeks post-STZ treatment in rats [83]. These rats also manifested severe loss of photoreceptors at 12 and 24 weeks, [83] while in WBN/ Kob rat retinas, photoreceptor degeneration occurs earlier, at four weeks of age [50]. Our recent study also confirmed RGC function loss and cell death in STZ-induced hyperglycemic mice at 32 weeks post-injection [84] and tree shrews at 16 weeks post-injection [31]. In addition to retinal neurons, RPE degeneration was reported in diabetic retinas. Thus, in four-month-old diabetic BB rats, hyperglycemia induces RPE degeneration through focal necrosis [85]. In hyperglycemic OLETF rat retinas, the decrease in the thickness of the RPE layer along with a reduction in the INL and ONL thicknesses occurs later, at nine months after induction of hyperglycemia [53]. Much later, at 50 weeks post-hyperglycemia induction, retinal detachment and fibrous proliferation occurs in Torii (SDT) rats with spontaneous diabetes [54]. In other model of spontaneous diabetes, ZFD rats, extensive glial activation along with photoreceptor outer segment (POS) degeneration occurs in 32-week-old retinas [86].The latter agrees with multiple studies demonstrating the thinning of the INL and IPL in OIR rat pups at P18 [67,84,87,88]. In addition, the thinning of the inner limiting membrane (ILM) is observed in STZ-induced SD retinas [85].

In STZ-induced diabetic mice, RGC loss occurs between 6 and 12 weeks [89]. RGC death occurs through apoptosis. The number of RGC apoptotic positive cells measured by TUNEL is 25% higher than that in control retinas [90]. These data are similar to our observation of about a 30% RGC death with this model, [84] although another study reported that the RGC density across the retina varies at 20 weeks post-STZ treatment [91]. A few studies with Ins2^Akita^ mice detected early cone photoreceptor cell loss at three months. The authors observed a significant reduction in the IPL and INL thicknesses along with a diminishing number of RGCs at 22 weeks and 36 weeks of hyperglycemia [42,92]. Similarly, the OCT analysis of 16- and 28-week-old diabetic db/db mice retinas showed thinning in the NFL and RGC layer at a rate of 0.104 μm per week, resulting in a reduction of the total retinal thickness by 28 weeks [91,93]. The 28-week-old diabetic db/db mice also showed TUNEL positive photoreceptor cells and reduction in the ONL thickness. STZ-induced hyperglycemia in mice also leads to GFAP overexpression in retinal astrocytes at five weeks post-STZ treatment, while Müller cell gliosis are not seen even after 15 months of DM [71,94]. In contrast, the OIR mice demonstrated a reduction in the total retinal, INL, and IPL thicknesses, as well as distorted photoreceptor OS, neuronal loss, hyperactivity of Müller cells, and microglial activation at P18 [66]. Our experiments with OIR pups confirmed these findings [84].

#### 3.1.2. The Detection of Functional Changes of the Neural Retina in Diabetic Rodents

Several studies with diabetic rats have reported ERG findings. First, there is a delay in the implicit time detected at four to seven weeks post-STZ. Second, a decrease in the a-wave of the scotopic ERG amplitude was detected at 10 weeks, while the b-wave amplitude was found to be reduced at 25 weeks after the induction of hyperglycemia [81,95,96,97]. Similar ERG findings were observed in SDT rats at 44 weeks post-STZ treatment [98,99] and mice and rats with proliferative retinopathy at P18 [66,67,87,88]. In the STZ-treated mice, retinal functional test showed a decrease in the implicit time for OP at 4-6 weeks, reduction in the scotopic ERG a and b-wave amplitudes at six months and diminished photopic ERG negative amplitudes at eight months after the induction of hyperglycemia [84,100,101,102]. Moreover, db/db (Lepr ^db^), Ins2^Akita^, and high fat diet-induced diabetic mice manifested similar retinal function changes detected by ERG at 6, 9 and 12 months, respectively [39,69,92,103].

### 3.2. Cellular Signaling Changes in the Diabetic Rodent Retina

#### 3.2.1. Insulin Signaling in the Diabetic Retina

Basal insulin receptor (IR) signaling has been extensively studied in the STZ-induced diabetic SD rat retina. It has been observed that the phosphorylation of insulin receptor (IR) in hyperglycemic retinas remained unchanged up to eight weeks post-injection, whereas PI3K activity was reduced by 25% compared to the controls. At 12 weeks post-STZ injection, both kinase activity and auto-phosphorylation of the IR were significantly decreased, suggesting that the basal IR activity is diminished in the diabetic retina. It was also demonstrated that Akt1 kinase activity was significantly diminished at eight weeks post-STZ injection, suggesting compromised glucose flux [104]. Kondo and colleagues observed important differences in insulin signaling between STZ-induced hyperglycemic mice and db/db mouse models developing DR. Specifically, IR expression and tyrosine phosphorylation were upregulated the first week post-STZ treatment in mouse retinas, but no changes were observed in 8- to 10-week-old db/db mice. Moreover, IRS-1 expression was unaltered, while IRS-2 expression was increased in both db/db and STZ-induced diabetic mouse retinas. In contrast, a few studies have reported a reduction in IR phosphorylation and an increase in the activity of the protein tyrosine phosphatase-1B (PTP1B) in the rod’s inner segments one-week post-STZ injection in mice [105]. An analysis of phosphorylated PTP1B in these mouse retinas point to PTP1B as a promising therapeutic target to delay neurodegeneration in diabetic retinas [106]. Reduced IR kinase activity at 12 weeks of hyperglycemia was also reported in a study with Ins2^Akita^ mice [42]. The potential contribution of excess glucose to local impairment of retinal insulin receptors and AKT activity has been proposed [104]. Our recent study confirmed an excess of glucose in diabetic retinas [84]. Moreover, other studies have reported reduced AKT phosphorylation as an early event in diabetic retinas with T1D [104,107] and T2D [108,109].

#### 3.2.2. Unfolded Protein Response (UPR) and Inflammation in the Diabetic Retina

Endoplasmic reticulum (ER) stress is one of the important features of the molecular pathobiology of the diabetic retina. Three independent UPR arms became activated during the ER stress response in diabetic retinas, including PKR-like ER kinase (PERK) and eukaryotic translation initiation factor 2α (eIF2α); inositol-requiring protein 1α (IRE1α)-X-box binding protein 1 (XBP1); and activating transcription factor 6 (ATF6)[110]. Activation of PERK kinase signaling results in phosphorylation (p) of eukaryotic translation initiation factor 2α (eIF2α), leading to global translational arrest and upregulation of activating transcriptional factor 4 (ATF4), C/ERB homologous protein (CHOP), and tribbles homolog 3 (TRIB3) proteins. The triggering of ATF6 is associated with its autophosphorylation, translocation to Golgi, and cleavage leading to active p-ATF6 transcriptional factor. After autophosphorylation, IRE1α, possessing both the RNAse and kinase activities, trims the Xbp1 transcriptional factor, leading to the formation of activated transcriptional factor, which controls a variety of gene expressions. Cellular stresses such as hypoxia and glucose imbalance can trigger UPR. ER stress markers are upregulated in diabetic rat retinas as early as eight weeks after the onset of diabetes induced by STZ in SD rats [111]. Apoptotic protein caspase 12, CHOP, and phosphorylated c-Jun N-terminal kinase 1 (MAPK) were dramatically upregulated in these retinas. In addition, the elevation of MAPK kinase was detected in RGCs [111]. However, the differences in the expression of the upstream and downstream mediators of PERK signaling, *Grp78* and *Atf4* genes, respectively, were not significant in this study. These findings suggest that AFT4 might not be the only signaling molecule responsible for the increased VEGF level in diabetic retinas [112]. Using immune-histochemical detection, the investigators reported the elevation of HIF-1α, ATF6, XBP1, and CHOP proteins in STZ-induced diabetic rat retinas at two and four months [82]. This elevation was accompanied by a decrease in the autophagy marker LC3B-II levels, indicating a potential reduction in autophagy in the diabetic retinas of mice with four months of hyperglycemia [82]. Pro-apoptotic BAX was detected in hyperglycemic ZFD rat retinas at six weeks of age [113].

Inducing diabetes and DR by STZ injection in mice, Chung et al. and Zhong et al. reported the activation of the ER stress response and pro-inflammatory signaling [114,115]. They found that the diabetic mouse retinas manifest increased expression of GRP78, pPERK, CHOP, VEGF, and peIF2α four weeks after STZ-induced hyperglycemia. Moreover, ATF4 deficiency resulted in altered inflammatory gene expression [115]. In addition to UPR markers, the above-mentioned study reported that MCP-1 and TNF-α expression simultaneously increased in diabetic retinas during the four-week period [114]. Interestingly, this study also highlighted that UPR signaling could be resolved later in diabetic mouse retinas, at six weeks post-STZ injection. In contrast, genetically modified Akita mice demonstrated an increase in p-elF2α and GRP78 proteins (PERK arm) in addition to elevated IRE-1 and TNFα expression at 12 weeks of age [116,117]. Elevated levels of GRP78, ATF4, and peIF2α were also found in the OIR model at P15 [117,118] and in 15-month-old db/db (Lepr ^db^) mice [119]. Moreover, our study showed that limiting ATF4 expression in hypoxic retinas significantly reduced the degree of neovascularization in the OIR mouse retinas, [118] and the deficiency in ATF4 could reduce IL-1β in diseased retinas [120].

**Figure 1:**
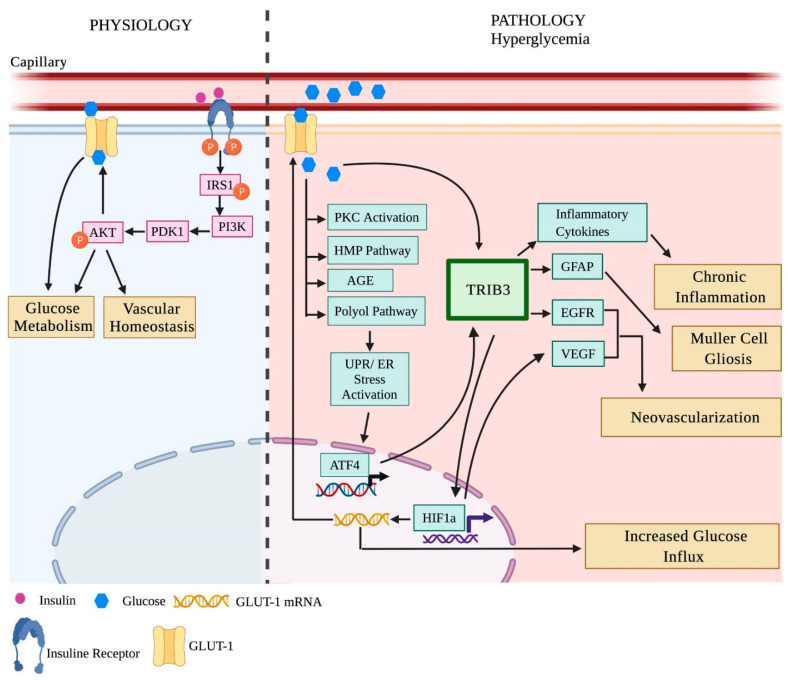
Tribbles homolog 3 (TRIB3) protein controls the development and progression of diabetic retinopathy. The PERK UPR marker TRIB3 is a known psuedokinase that binds and prevents AKT phosphorylation by PDK1. In addition, it controls the expression of HIF1α, EGFR, GFAP, and inflammatory cytokines in cells. In hyperglycemic retinas and retinas of mice with proliferative retinopathy, TRIB3 is significantly upregulated. This results in overexpression of HIF1α, EGFR, GFAP, and inflammatory cytokines (Icam1, Nf-kb1, Rc3h1, Zc3h12a, VEGF, COX2, and AIF1, [84]). In turn, overexpressed HIF1α leads to GLUT1 activation and, together with TRIB3, increases the influx of glucose, which affects the overall glucose metabolism in diabetic retinas. Aberrant glucose flux and hyperglycemia in diabetic retinas are responsible for the activation of PKC, HMP, AGE, and polyol pathways, which eventually leads to chronic UPR activation. TRIB3-mediated pro-inflammatory cytokine expression results in chronic inflammation, GFAP increase leads to the retinal gliosis observed in proliferative retinas, and VEGF elevation triggers neovascularization in the late stages of DR. Image created by Biorender.com, (accessed on 30 May 2021).

Our recent study with diabetic TRIB3 KO demonstrated that TRIB3 is a master regulator of insulin signaling and glucose metabolism in the retina (Figure 1). Thus, we revealed that TRIB3 is induced in diabetic retinas, leading to overexpression of HIF1a, GFAP, VEGF, GLUT1, and EGFR proteins. In turn, HIF1a regulates GLUT1 expression and, together with TRIB3, controls the uptake of glucose in the retina. Moreover, TRIB3 mediates the retinal ganglion cell fate decision, while TRIB3 KO results in neuronal survival and improvement of vascular health.

Changes in inflammatory gene expression across varied rodent models of DR have also been reported. For example, six-week-old ZFD rat retinas manifest an increase in the levels of TNF-α and NF-kB [113]. Inflammatory proteins such as clusterin, the tissue inhibitor of metalloproteinase (TIMP)-1, β-2 microglobulin, and von Willebrand factor were overexpressed in the SD rat retinas at four weeks and, particularly, at three months post-STZ injection. In addition, the overexpression of fibroblast growth factor-2 (FGF2) was detected in the ONL of diabetic rat retinas at three months post-STZ. It is also worth mentioning that inflammatory changes are strain-dependent in diabetic rat models. For example, compared to Long-Evans and Brown Norway rats, SD rats show inflammatory changes more similar to those found in human diabetic retinopathy [121]. SD rat pups with OIR were also reported to overexpress inflammatory markers at P16 [58,64].

Overall, the above-mentioned studies emphasize that alterations in cellular molecular signaling often precede retinal pathophysiological events. These findings suggest that dysfunctional insulin signaling, ER stress response, and inflammation are involved in the pathological progression of DR and can be targeted to develop novel cellular therapies for DR (Table 2).

## 4. Non-Rodent Models of Diabetic Retinopathy

The induction of hyperglycemia and the development of DR in dogs are very often achieved with a high-galactose diet. These treated dogs develop DR pathology changes similar to those found in patients with DR. It is worth mentioning that dogs with STZ-induced diabetes leading to DR were the first animal models to develop both NPDR and PDR [122]. In these dogs’ retinas, the researchers detected an NPDR marker—pericyte loss—at nine months post-STZ injections. PDR complications such as hemorrhages, microaneurysms, basement membrane (BM) thickening, vitreous detachment, and neovascularization usually develop in this model later—within 28 to 68 months post-STZ treatment [33,122].

Diabetic swine are another model of DR. The retinas of these animals have many similarities to human retinal tissues. Alloxan-induced T1D and diet-induced T2D pig models are frequently used models of DR. Alloxan treatment leads to the development of pericyte loss and BRB breakdown at 20 weeks after hyperglycemia in these pig’s retinas [34]. In contrast, another study found that Alloxan induces Müller cell contraction-promoting activities affecting the vitreous at 30 days after induction of hyperglycemia in the swine model [123]. These findings suggest that the swine Müller cell contraction-promoting activity resulting in retinal detachment at the advanced stages of diabetes is similar to the changes seen in humans with DR. Another swine model is the combination model of DM and hypercholesteremia (DMHC). This model demonstrated increased BRB permeability, gliosis, microglial activation, and decreases retinal thickness at 24 weeks [124]. Kleinwort et al. reported intraretinal microvascular abnormalities and central retinal edema observed in a swine model of DR two years after the onset of hyperglycemia [125]. In four-month-old Ossabaw pigs fed with a western diet (high fat/high fructose corn syrup/high calories), Lim et al. reported development of chronic DM at 10 weeks. These animals also manifested retinal INL disruption, thickened BM, the formation of pericyte ghosts and acellular capillaries, and an increase in fibronectin expression at the age of six months [126].

The major drawback of DR studies on large animals is the delay in the development of histological features, making large animals less attractive species. Hatchell and colleagues were the first to develop the feline diabetic model using pancreatectomy and monitor them for nine years with regular checks for hyperglycemia every six months. They reported the presence of thickened BM at three months, microaneurysms at five years, and neovascularization around 6.5–8 years post-surgery in the diabetic cat retina [37]. Rhesus monkeys can develop DR through the implantation of 100 µg of human recombinant VEGF [59] or through STZ-induced hyperglycemia. Retinal pathobiology associated with diabetes in these animals were observed after six years of hyperglycemia [127].

In addition to large diabetic models, small non-rodent animals manifest some features of DR after induction of hyperglycemia. For example, tree shrews are closely related to the primate animals that have cone-dominant retinas. Recently, our group validated tree shrews as a novel model of T1D manifesting some features of diabetic retinopathy [31]. Thus, we propose that diabetic tree shrews develop the retinal phenotypes of cone photoreceptor degeneration and RGC dysfunction, and mimic early stages of hyperglycemia-induced DR. Besides the fact that this model lays the groundwork for better understanding molecular pathophysiology of DR in humans, it can be an ideal bridge between the non-human primate and rodent models of diabetes; it simulates critical pathophysiological aspects of human DR and could be used to evaluate the effect of systemic pathogenesis of human diabetes, including the affected pancreas, liver, and kidneys on the development and progression of DR. A rabbit model was generated by STZ injections, resulting in the development of DR. Forty percent of diabetic rabbits with an average BGL of 200 mg/dL develop retinopathies after 135 days of initial STZ injections. Zebrafish von Hippel-Lindau (vhl) mutants and transgenic zebrafish (Fli-EGFP-Tg) exposed to hypoxic conditions also manifest features of DM [60,61].

### 4.1. Pathological Changes in Non-Rodent Animal Models of Diabetic Retinopathy

After 135 days of initial STZ injections, hyperglycemic rabbits developed high-incident DR, classified as serious vasculopathy with retinal hemorrhages, vascular lesions, and venous thrombosis, as well as more severe DR, classified as PDR [29]. In another rabbit model of angiogenesis generated by the implantation of human recombinant VEGF at a dose of 30 µg, the vitreous showed abnormal tortuous blood vessels followed by vascular leakage at 14 days and neovascularization in the retina at 21 days of transplantation [59]. In zebrafish exposed to hypoxia, the retina demonstrated new sprouts in the optic capillary plexus and the formation of capillary tips 12 days post-exposure [60]. Aberrant blood vessel formation and an increase in VEGF mRNA expression was reported in a genetic fish model of DR, the mutant *vhl* zebrafish, at two days post-fertilization [61]. Zebrafish immersed in a high-glucose solution over a period of 30 days showed irreversible reduction in the IPL and INL of the retina and thickening of the blood vessels and their basement membrane [128,129]. The genetic manipulation and easily achievable hypoxic conditions with this model favors the use of zebrafish as a model for DR.

Primates closely mimic the disease pathology of the human retina. The implantation of human recombinant VEGF in the rhesus monkey resulted in an increase in vascular permeability, a breakdown of BRB, and the tortuosity of the blood vessels in the vitreous at two to three weeks post-treatment [59]. STZ-induced diabetes leads to retinal pathology after six years of hyperglycemia in monkeys that include the presence of cotton-wool spots, macular atrophy, arteriolar occlusion, focal intraretinal capillary leakage, and capillary dilatation [127]. In addition to STZ-induced hyperglycemia, the markers of DR were also observed in mild hypertensive rhesus monkeys. Retinopathy in these animals was categorized into three stages: 1) microvascular abnormality with capillary dropout, 2) vascular leakage, intraretinal exudates, and cystoid degeneration with cotton wool spots, and 3) vascular occlusion and retinal atrophy [40]. In obese monkeys with T2D, these retinal findings develop slowly over a period of 1.25 to 15 years [130]. With aging, diabetic rhesus monkeys with T2D demonstrated a decline in retinal function measured by mERG recording; the a-wave of the scotopic ERG and the oscillatory potential were reduced in animals aged over five years [30]. Interestingly, the development of DR in monkeys could be achieved more quickly. For example, a recently developed diet-induced primate model of the marmoset monkey manifested development of DR within 2.5 years. Marmoset monkeys, which are smaller than rhesus macaques and easier to maintain, develop diabetes-induced retinal phenotype faster than other monkey models and thus present an excellent animal model of DR [38]. Table 3 summarizes major pathological changes observed in non-rodent models of diabetic retinopathy.

### 4.2. Cellular Signaling Changes in the Diabetic Retina of Non-Rodent Models

It is worth mentioning that the reports on the alteration of cellular signaling in non-rodent diabetic retinas are scarce in the PubMed system. Thus, it has been demonstrated that high glucose triggers the *Vegf, Il-6*, *Il-1β*, *Stat3*, and *Tnfα* mRNA expression in Zebrafish retinas that were associated with an increase in TUNEL^+^ cells in the retina. [131] Rabbits with T1D mellitus induced by alloxan injection and analyzed at six weeks after induction of diabetes manifest an increase in the oxidated proteins and lipids, a decrease in enzymatic activity (catalase, glutathione peroxidase and superoxide dismutase), and a decline in p-PI3K/PI3K, p-AKT/AKT and p-GSK3/GSK3 ratios [132]. In contrast, our study with diabetic tree shrews demonstrated that diabetic retinas experience the elevation of TRIB3 and p-AKT/AKT→ p-mTOR/mTOR signaling, whereas the IRS level at 16 weeks of hyperglycemia was reduced [31]. Although retinal vascular leakage was not observed in these animals, the retina VEGF level was slightly increased, suggesting that vascular abnormalities could be detected later in diabetic tree shrews.

## 5. The Limitation of Animal Models of Diabetic Retinopathy

**Figure 2:**
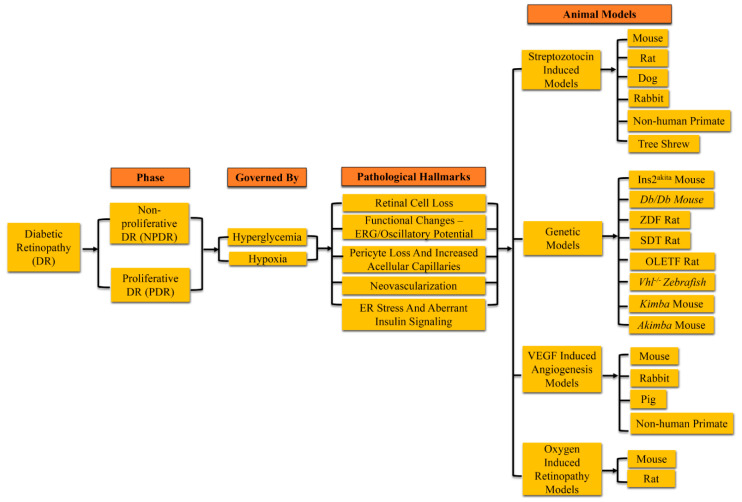
Animal models of diabetic retinopathy were developed to mimic non-proliferative and proliferative stages of diabetic retinopathy (DR). These models were created by inducing hyperglycemia or hypoxic conditions. Observed retinal pathophysiological features present neuronal function and cell loss in addition to vascular dysfunction. These models include rodents, cats, dogs, pigs, non-human primates, and zebra fish. They are genetically modified and/or pharmacologically-induced animal models of DR. (ERG-Electroretinogram; ER- Endoplasmic reticulum; ZDF- Zucker diabetic fatty; OLETF- Otsuka Long-Evans Tokushima fatty; vhl- von Hippel-Lindau).

In this review, we highlighted the differences between various models of DR (Figure 2). The cellular molecular pathology associated with the long-term development of diabetes seems not to be uniform across different research groups. For example, Martin and colleagues reported retinal cell loss in STZ-treated mouse retinas, while Feit-Leichman and co-authors observed no changes in the retinas of these diabetic mice [71,90]. These findings could be attributed to the different strains used for the development of STZ-induced hyperglycemia. In addition, although high BGL is consistent across the studies, investigating groups may use additional injections of STZ to maintain high BGL in experimental animals. Interestingly, STZ-induced hyperglycemia is gender dependent. Male mice are more prone to STZ-induced pancreatic damage compared to females because the effect of STZ is inhibited by the female hormone estrogen [133]. Saadane and colleagues recently reported that increasing the STZ dose by almost 36% yields hyperglycemia levels and subsequent retinal pathological changes in females similar to those found in males [134]. Despite these limitations, the chemical induction of hyperglycemia is still the fastest method to induce diabetes compared with spontaneous T1D and T2D models. The OIR mouse model is a particularly strain-dependent model, and the maintenance of genetic background in experimental mice is of the utmost importance [65]. The rodent models, in comparison to large mammals, provide better accessibility to molecular changes and genetic manipulation, as well as a shorter time period for the development of diabetes and easier handling. The only advantage of using primates and other large animals is their close resemblance to human retinal physiology. Thus, choosing the appropriate animal model for DR research is a challenging process and requires careful evaluation.

## 6. Conclusions

The current review emphasizes several critical factors that should be considered before making a decision on a suitable animal model of DR. In particular, factors such as the duration of the study, the methods of induction of hyperglycemia, and the established molecular and pathophysiological markers of diabetic retinopathy should be considered carefully. Because not all animal models accurately mimic the diabetic human retinal pathology, it is important to evaluate the strengths and weaknesses of each animal model to properly design research experiments.

## Figures and Tables

**Table 1:** Current animal models of diabetic retinopathy.

	Hyperglycemia Induction				
	Method	Species	Dosage	Hyperglycemia	References
1.	Streptozotocin (STZ)	mouse, rat, rabbit, tree shrew, monkey, cat	mouse and rat—intraperitoneal (IP) 40–80 mg/kg (5 days), mouse—IP 150–200 mg/kg (single dose), rat—IP 30–80 mg/kg (single dose), rabbit—intravenous (IV) 110 mg/kg (single dose), tree shrew—IP 80 mg twice a week apart and IP 175 mg/kg (single dose).	mouse and rat approx. 1-week post-STZ	[24,25,26,27,28,29,30,31]
2.	Alloxan	mouse, rat, rabbit, swine, dog	rat-IP 80–140 mg/kg (single dose), rat-subcutaneous (SC) 80–120 mg/kg (single dose), dogs-IV 50 mg/kg (single dose).		[27,32,33,34]
3.	Pancreatectomy	cat, dog			[35,36,37]
4.	High galactose /fat type 2 diet	mouse, rat, dog, swine, zebrafish, monkey			[22,35,38,39,40]
	**Spontaneous Hyperglycemia**				
	Mouse			Hyperglycemia	
1.	Ins2Akita mouse: Type I Diabetes Mellitus (DM), mutation in insulin			4 weeks	[41,42]
2.	Non-obese mouse (NOD): Type I DM, autoimmune model			12 weeks	[43,44]
3.	db/db (Leprdb) mouse: Type II DM			8–10 weeks	[45]
4.	Kimba mouse:Transgenic mouse (tr029VEGF)				[46]
5.	Akimba mouse: Ins2^Akita^ /VEGF ^(+/−)^			4 weeks	[47]
	Rat			Hyperglycemia	
1.	Biobreeding rats: Type I DM, autoimmune model			3 months	[48,49]
2.	Wistar Bonn/Kobori (WBN/Kob) rats: Type II DM			9 months	[50]
3.	Zuker diabetic fatty (ZDF) rats: Type II DM			5–10 weeks	[51,52]
4.	Otsuka Long-Evans Tokushima fatty (OLETF) rats: Type II DM			5 months	[53]
5.	Spontaneous diabetic torii (SDT) rats: Type II DM			5 months	[54]
	**Neovascularization**				
	Mouse				
1.	Oxygen induced retinopathy (OIR)				[55]
2.	Kimba mouse				[46]
3.	Akimba mouse				[47]
	Rat, Canine				
1.	Oxygen induced retinopathy (OIR)				[56,57,58]
	Rabbit				
1.	Implantation of human recombinant VEGF in the vitreous				[59]
	Zebrafish				
1.	Angiogenesis				[60,61]
	Monkey				
1.	Implantation of human recombinant VEGF in the vitreous				[59]

**Table 2:** Cellular signaling, loss of retinal function and integrity in rodent models of diabetic retinopathy.

Molecular Signaling				
	Model	Changes	Duration of Hyperglycemia	References
1.	STZ Rat	Elevated CHOP, Caspase 12, MAPK retinal cytokines	8 weeks	[82,104,111,121]
		Reduced IR kinase activity	8 weeks	
		Elevated retinal cytokines	3 months	
		Reduced IR kinase activity and autophosphorylation and downregulation of IRS-2 & PI3K	3 months	
		Upregulation of HIF-A, ATF-6, XBP1	4 months	
2.	ZFD Rat	Elevated Bax, TNF-α and NF-kappaB	6 weeks	[113]
3.	OIR Rat	Elevated VEGF, PDEG and TNF-α	P16	[58,64]
4.	STZ Mouse	Upregulation of GRP78, pPERK, CHOP, VEGF, pEIF2α, retinal cytokine and TNF-α	4 weeks	[84,105,106,114,115,116,117]
		Elevated IR expression and tyrosine phosphorylation; upregulated IRS-2 and reduced PDK1/ AKT protein levels and phosphorylation	1 week	
		Reduced IR phosphorylation	1 week	
		Upregulation of TRIB3 and inflammatory cytokines (Icam1, Nf-kb1, Rc3h1, Zc3h12a, VEGF, COX2, and AIF1)	4 weeks	
5.	Ins2Akita Mouse	VEGF and TNF-α elevation, increased mRNA expression; protein expression of GRP78 and elevated peIF2α and ATF4 and reduced IR kinase activity	12 weeks	[42,116,117]
6.	Leprdb (db/db) Mouse	Increased IRS-2 expression and reduced PDK1/ AKT protein levels and phosphorylation	10 weeks	[116,119]
		GFAP activation, increased expression of HIF-A, VEGF, GRP78, p-IRE-1, CHOP, Casapase-3 and ATF4	15 months	
**Microangiopathy**				
	**Model**	**Changes**	**Duration of** **Hyperglycemia**	**References**
1.	STZ Rat	Blood retinal barrier disruption	2 weeks	[8,72,74]
		Adherent leukocytes	8 weeks	
		Thickened Basement Membrane (BM)	12 weeks	
		Neovascularization	3–4 months	
2.	Alloxan Rat	Leukocytosis	2 months	[75,76]
		Neovascularization	9 months	
		Pericyte loss, acellular capillaries, and BM thickening	12 months	
3.	BB Rat	Basement membrane thickening	4 months	[48,49,77]
		Blood retinal barrier breakdown	6 months	
		Pericyte loss	8 months	
4.	ZDF Rat	BM thickening, pericyte loss and acellular capillaries	6 months	[51,52]
5.	OLETF Rat	BM thickening, pericyte loss and acellular capillaries	9 months	[53,78]
6.	OIR SD Rat	Increased extra retinal neovascularization and impaired pericyte distribution	P18	[67]
7.	STZ Mouse	Increased vascular permeability	8 days	[26,70,71,84]
		Decreased arteriolar diameter and velocity	8 weeks	
		BM thickening	4–15 months	
		Pericyte loss, acellular capillaries and pericyte ghost	6–9 months	
8.	Ins2Akita Mouse	Leukocytosis	8 weeks	[42,69]
		Increased vascular permeability	12 weeks	
		Blood vessels in the outer plexiform layer (OPL) and microaneurysms	6 months	
		Acellular capillaries, BM thickening and neovascularization.	9 months	
9.	Kimba Mouse	Abnormal blood vessel development around photoreceptor	P28	[46,68]
		Increased vascular permeability and adherent leukocytes	6 weeks	
		Loss of retinal capillaries, neovascularization, increased avascular area and alteration in the vessel length	9 weeks	
		Pericyte loss	24 weeks	
10.	Akimba Mouse	Microaneurysms, neovascularization, blood vessel constriction, beading, vessel edema, capillary dropout, and new vessel formation it the ONL	8 weeks	[47]
11.	OIR Mouse	Irregular blood vessel development and reduced inner retinal plexus and deep plexus	P18	[66]
12.	Db/db Mouse	Increased vascular permeability and BM thickening	13–14 weeks	[79,80]
		Pericyte loss	18 weeks	
		Acellular capillaries	26 weeks	
13.	High-fat diet Mouse	Pericyte loss, blood retinal barrier disruption and vascular leakage	12 months	[39]
**Retinal Integrity**				
	**Model**	**Changes**	**Duration of Hyperglycemia**	**References**
1.	STZ Rat	Decreased pre- and post-synaptic photoreceptor ribbon synapses	4 weeks	[81,82,83]
		Increased GFAP reactivity	6–7 weeks	
		Loss of ONL, INL, GCL	12–16 weeks	
		Severe photoreceptor cell loss	24 weeks	
2.	WBN/Kob Rat	Photoreceptor degeneration	4 weeks	[50]
		Severe OS and ONL degeneration	5–14 months	
3.	BB Rat	RPE degeneration	4 months	[85]
4.	ZDF Rat	Decreased OS, damage to amacrine cells and RPE with gliosis	32 weeks	[86]
5.	OLETF Rat	Decreased INL and photoreceptor cells	9 months	[53]
6.	OIR Rat	Reduction in OS, INL, IPL, total retinal thickness, astrocytes and increased muller activity	P18	[67,87]
7.	High galactose Rat	Increased gliosis and reduced INL and OPL	28 months	[85]
8.	STZ Mouse	GFAP hyperactivity	5 weeks	[71,84,89,91,94]
		Reduced ONL, INL thickness	6–14 weeks	
		Total retinal thickness reduced	20 weeks	
		No retinal cell loss and gliosis	8–12 months	
		Reduced RGCs	8 months	
9.	Ins2Akita Mouse	GFAP hyperactivity	8 weeks	[42,92]
		Reduced IPL, INL and cone photoreceptors	3 months	
		Reduced RGCs	22 weeks	
		Decreased presynaptic and post-synaptic photoreceptor ribbons	36 weeks	
10.	db/db Mouse	Reduced NFL and RGCs	16-28 weeks	[91,93]
		Reduced total retinal thickness	28 weeks	
11.	Akimba Mouse	Photoreceptor cell death	28 weeks	[47]
12.	OIR Mouse	Total retinal thickness reduction, distorted photoreceptor OS, neuronal loss, hyperactivity of Müller cells, microglial activation and disrupted INL and IPL	P17-188	[66,84]
**Retinal Electrophysiology**				
	**Model**	**Changes**	**Duration of** **Hyperglycemia**	**References**
1.	STZ Rat	Decrease in OP amplitude	2–7 weeks	[81,98,99]
		Decrease in OP implicit time	7 weeks	
		Decreased a- and b-wave amplitude	10–12 weeks and at 44 weeks	
2.	OIR Rat	Decreased a- and b-wave amplitude	P18	[67,87,88]
3.	STZ Mouse	Reduced OP amplitude and implicit time	4–6 weeks	[84,100,101,102]
		Reduced a- and b-wave amplitude	6 months	
		Reduced PhNR amplitude	8 months	
4.	Ins2Akita Mouse	Decreased OP amplitude, delay in the OP and decreased b-wave	9 months	[42,92]
5.	Db/db Mouse	Delay in the b-wave, delay in the OP implicit time and decreased amplitude of both photopic and scotopic b-wave	16–24 weeks	[93,103]
6.	OIR Mouse	Significant decrease in the amplitude of a- and b-wave	P18	[66]
7.	High-fat diet Mouse	Decreased OP amplitude	12 months	[39]

**Table 3:** Pathological changes in non-rodent models of diabetic retinopathy.

	Model	Pathological Changes	Induction of DR	References
1.	VEGF-induced angiogenesis Rabbit	Tortuous blood vessels	2 weeks	[59]
		Vascular leakage		
		Neovascularization	3 weeks	
2.	Alloxan Rabbit	Increase in the oxidated proteins and lipids	6 weeks	[132]
		Decline in p-PI3K/PI3K, p-AKT/AKT and p-GSK3/GSK3 ratios		
3.	STZ Rabbit	Retinal hemorrhages and venous thrombosis	19 weeks	[29]
		Vascular lesions		
4.	Hypoxic Zebrafish/vhl-mutant Zebrafish	Aberrant blood vessel formation	2-days-post-fertilization (dpf)	[60,61]
		Formation of capillary tips and sprouts in optic capillary plexus	12 dpf	
		Increased mRNA VEGF		
5.	Hyperglycemic Zebrafish	Increased Vegf, Il-6, Il-1β, Stat3, and Tnfα mRNA expression	3-6 dpf	[131]
		Reduction in the IPL and INL	30 dpf	[128,129]
		Thickening of the blood vessels		
		BM thickening		
6.	VEGF induced angiogenesis-Primate	Vascular permeability	2–3 weeks	[59]
		Breakdown of BRB		
		Tortuosity of the blood vessels		
7.	STZ Primate	Presence of cotton-wool spots	6 years	[127]
		Macular atrophy		
		Arteriolar occlusion		
		Focal intraretinal capillary leakage		
		Capillary dilatation		
8.	Obese Primate	Decreased a-wave of the scotopic ERG	5 years	[130]
		Reduced oscillatory potential		
9.	Diet-induced DR Marmoset	Excess vascular permeability.	2.5 years	[38]
		Increased acellular capillaries and pericyte loss		
		BM thickening and vessel tortuosity		
		Thickening of the retinal foveal		
		Microaneurysms		
10.	STZ Tree shrew	Upregulation of TRIB3	16 weeks	[31]
		Upregulation of p-AKT/AKT→ p-mTOR/mTOR		
		Increased IRS		
		RGC function loss and cell death		
11.	STZ Dog	Pericyte loss	9 months	[33,122]
		Hemorrhages and microaneurysms	28–68 months	
		BM thickening		
		Vitreous detachment		
		Neovascularization		
12.	Pancreatectomy Cat	BM thickening	3 months	[37]
		Microaneurysm	5 years	
		Neovascularization	6.5–8 years	
13.	Alloxan Pig	Pericyte loss and BRB breakdown	20 weeks	[34]
14.	STZ Pig	BRB permeability	24 weeks	[124]
		Gliosis and microglial activation		
		Decrease in retinal thickness		
15.	High-fat diet Pig	INL disruption	6 months	[126]
		BM thickening		
		Pericyte ghosts and acellular capillaries		
		Increase in fibronectin expression		

## Data Availability

Not applicable.
